# Novel biallelic *COL25A1* variants broaden the clinical spectrum from congenital cranial dysinnervation disorders to fetal lethal phenotypes

**DOI:** 10.1038/s41431-025-01839-4

**Published:** 2025-03-29

**Authors:** Frederike L. Harms, Christian Müller, Fanny Kortüm, Maja Hempel, Malik Alawi, Maha S. Zaki, Rasha M. Elhossini, Mohamed S. Abdel-Hamid, Lama AlAbdi, Fowzan S. Alkuraya, Wesam Kurdi, Tristan Celse, Marta Spodenkiewicz, Tiphany Laurens, Klaus Dieterich, Sujatha Jagadeesh, Sandesh Salvankar, Katta M. Girisha, Kerstin Kutsche

**Affiliations:** 1 Institute of Human Genetics, University Medical Center Hamburg-Eppendorf, Hamburg, Germany; 2 Bioinformatics Core, University Medical Center Hamburg-Eppendorf, Hamburg, Germany; 3 Clinical Genetics Department, Human Genetics and Genome Research Institute, National Research Centre, Cairo, Egypt; 4 Medical Molecular Genetics Department, Human Genetics and Genome Research Institute, National Research Centre, Cairo, Egypt; 5 Department of Translational Genomics, Center for Genomic Medicine, King Faisal Specialist Hospital and Research Center, Riyadh, Saudi Arabia; 6 Department of Obstetrics and Gynecology, King Faisal Specialist Hospital and Research Center, Riyadh, Saudi Arabia; 7 Service de Génétique Médicale, Centre Hospitalier Universitaire de La Réunion, Saint-Denis, France; 8 Universite Grenoble Alpes, Inserm, U1209, CHU Grenoble Alpes, Grenoble, France; 9 Medical Genetics, Institute of Advanced Biosciences, Grenoble, France; 10 Mediscan Systems, Chennai, India; 11 Suma Genomics Private Limited, Manipal, India; 12 Department of Medical Genetics, Kasturba Medical College, Manipal, Manipal Academy of Higher Education, Manipal, India; 13 Department of Genetics, College of Medicine and Health Sciences, Sultan Qaboos University, Muscat, Oman; 14 German Center for Child and Adolescent Health (DZKJ), partner site Hamburg, Hamburg, Germany; 15 Present Address: Institute of Human Genetics, Heidelberg University, Heidelberg, Germany

**Keywords:** Genetics research, Diseases

## Abstract

Biallelic variants in *COL25A1* have been associated with isolated congenital cranial dysinnervation disorders (CCDDs) and arthrogryposis multiplex congenital (AMC) with or without CCDD. *COL25A1* encodes collagen XXV that belongs to the subfamily of membrane-associated collagens with interrupted triple helices. COL25A1 contains four non-collagenous and three collagenous domains. Three alternatively spliced *COL25A1* transcript variants are known. In mice, Col25a1 is required for intramuscular motor innervation and cranial motor neuron development. We report seven subjects with novel biallelic *COL25A1* pathogenic variants, including three AMC-affected individuals, one of whom died in infancy, and four unrelated fetuses. We expand the associated phenotypic spectrum as fetuses showed lethal phenotypes including reduced or no movement, contractures, and hydrops in three and growth retardation and skeletal abnormalities in one. The molecular spectrum includes two microdeletions encompassing several 5′ or 3′ exons, two missense, one nonsense, one frameshift, and one variant affecting splicing. In fibroblasts of the subject who was compound heterozygous for the c.367G > C and c.1198G > T variants, we identified skipping of exon 3 in *COL25A1* mRNAs due to the G-to-C change. These aberrantly spliced transcripts were subject to nonsense-mediated mRNA decay. Analysis of transcriptome sequencing data from primary human fibroblasts without *COL25A1* pathogenic variants revealed novel *COL25A1* exon-exon junctions and 13 not previously annotated alternatively spliced *in-frame* exons. We hypothesized that interindividual variation in the splicing of *COL25A1* exons in different tissues may underlie the variable phenotypes in the affected individuals.

## Introduction

**Fig. 1:**
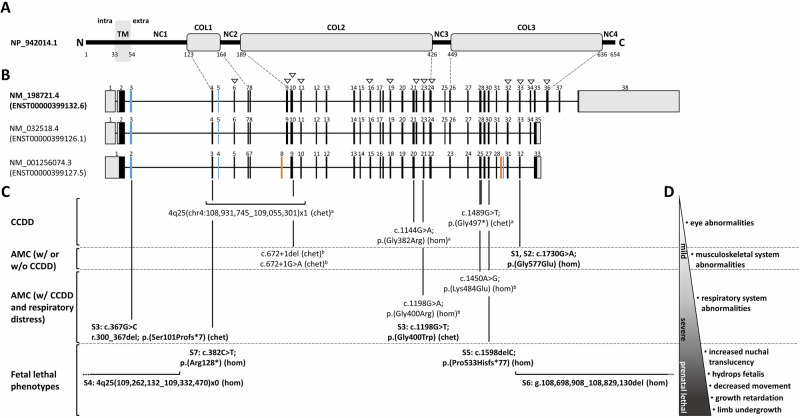
Collagen XXV domain structure, *COL25A1* transcript variants, biallelic *COL25A1* pathogenic variants, and associated clinical features. **A** Schematic representation of the domain structure of collagen XXV/COL25A1 (NP_942014.1; longest isoform). Amino acid numbering is given according to Fig. 3A in ref. [6]. Collagenous domains (COL1 to COL3) are represented by gray boxes and non‐collagenous domains (NC1-4) by black lines. The NC1 domain is composed of an intracellular (intra), a transmembrane (TM, shaded in gray), and an extracellular (extra) region. Dotted lines indicate the exons encoding the COL1-3 domains [according to NM_198721.4—see (**B**)]. **B** Schematics of *COL25A1* transcript variants in the NCBI and Ensembl databases (last accessed 10/2024), with the MANE select transcript NM_198721.4 highlighted in bold. Exons are represented by boxes, with the coding region in black (*in-frame* exons) or blue (*out-of-frame* exons) and untranslated regions in gray; introns are indicated by black lines. The numbering of selected exons is given. Alternatively spliced exons that are only present in transcript variant NM_001256074.3 are indicated in orange. Novel alternatively spliced exons identified in transcriptome sequencing data of 42 fibroblast cDNA samples are indicated by white arrowheads (see Supplementary Fig. S3). NM_001256074.3 is the predominant transcript variant expressed in fibroblasts. **C** Location of previously published and novel biallelic *COL25A1* variants (in bold) is shown below the exon-intron structure. Variant description is given according to the *COL25A1* mRNA reference number NM_198721.4. Associated phenotypes are shown on the left, arranged from mild at the top to severe at the bottom. **D** Phenotypic spectrum from mildest to most severe associated with biallelic *COL25A1* variants. The triangle visualizes the increasing severity of the phenotype from isolated ocular manifestations (white; mild phenotype) to complex fetal phenotypes (black; prenatal lethal). Associated clinical features are shown on the right. ^a^Variants previously published in patients with CCDD [3]. The deletion chr4:108,931,745–109,055,301 (GRCh38/hg38) [chr4:109,852,901–109,976,457 (GRCh37/hg19)] encompasses exons 4–11 and not only exons 4–10 as reported previously [3]; ^b^variants previously published in patients with AMC with or without CCDD [4]; AMC arthrogryposis multiplex congenita, C C-terminus, CCDD congenital cranial dysinnervation disorder, chet compound heterozygous, hom homozygous, MANE select matched annotation between NCBI (RefSeq) and EBI (Ensembl/GENCODE), N N-terminus, S subject, w/ with, w/o without.

Congenital cranial dysinnervation disorders (CCDDs) comprise a heterogeneous group of diseases that are characterized by defects in the innervation of the extraocular muscles. Affected individuals cannot fully move one or both eyes [1]. Biallelic *COL25A1* pathogenic variants have been associated with isolated CCDD. A homozygous *COL25A1* missense variant, p.(Gly382Arg), was detected in three affected siblings of one family, while a heterozygous nonsense variant p.(Gly497*) in trans with a heterozygous multiple-exon deletion in *COL25A1* was identified in a single patient of the second family [2, 3]. Recently, biallelic *COL25A1* pathogenic variants distinct from those reported previously have been identified in five individuals from three unrelated families with arthrogryposis multiplex congenita (AMC) [4] (Fig. 1), a heterogeneous group of congenital conditions characterized by joint contractures in two or more body areas [5]. Thus, the previously described phenotypic spectrum of *COL25A1*-related conditions ranged from isolated CCDD to AMC with or without CCD [4].

*COL25A1* encodes collagen XXV that belongs to the subfamily of membrane-associated collagens with interrupted triple helices (MACITs). MACITs can be cleaved by a furin convertase and therefore exist in membrane-bound and secreted forms. COL25A1 contains four non-collagenous (NC) domains (NC1-NC4) and three collagenous domains (COL1-COL3). The N-terminal NC1 is composed of cytoplasmic, transmembrane, and extracellular portions (Fig. 1) [6, 7]. Three *COL25A1* transcript variants have been characterized, including the longest transcript variant encoding the full-length collagen XXV (isoform 1) composed of 654 amino acid residues (MANE Select transcript NM_198721.4; ENST00000399132.6) and the shorter transcript variants NM_032518.4 (ENST00000399126.1; isoform 2 composed of 642 amino acid residues) and NM_001256074.3 (ENST00000399127.5; isoform 3 composed of 645 amino acid residues) (Fig. 1).

During mouse development, *Col25a1* is expressed in neurons in the spinal cord, including motoneurons, and in skeletal muscle cells [8, 9]. *Col25a1* knockout mice die in the early neonatal period due to respiratory failure indicating neuromuscular defects [9]. Studies in *Col25a1*-deficient mice and in mice with conditional *Col25a1* disruption in developing muscles demonstrate that the membrane-bound collagen is required for intramuscular motor innervation and the development of cranial motor neurons [9, 10]. Together, these data show an important role of Col25a1 in neuromuscular development in general and intramuscular motor innervation in particular [7, 10].

We here report on seven subjects with biallelic *COL25A1* variants, including two siblings and four fetuses, with a broad spectrum of clinical features, ranging from an AMC phenotype to fetal lethal conditions.

## Material and methods

### Genetic analyses

Genomic DNA was extracted from peripheral blood samples using standard procedures. Sequencing was performed by single exome sequencing (ES) in subjects 1 and 7, trio ES in subjects 3 and 4 with their respective parents, and trio genome sequencing (GS) in subject 6 and parents. Chromosomal microarray analysis was performed in subject 5 (for details see Supplementary Information). When necessary, *COL25A1* variants were validated and/or segregated by Sanger sequencing or quantitative PCR in the families (Supplementary Information and Supplementary Fig. S1). Primer sequences can be found in Supplementary Table S1.

### Cell culture

Primary dermal fibroblasts were cultured from a skin biopsy of subject 3 and one healthy female control (34 years old) in Dulbecco’s modified Eagle medium (DMEM; Thermo Fisher Scientific, Waltham, MA, USA) supplemented with 10% fetal bovine serum (GE Healthcare, Chicago, IL, USA) and penicillin-streptomycin (100 U/mL and 100 mg/mL, respectively; Thermo Fisher Scientific). The same passage number of subject and control fibroblasts was used in all experiments. Primary fibroblasts were regularly tested for mycoplasma contamination and confirmed to be mycoplasma-free. To inhibit nonsense-mediated mRNA decay, fibroblasts were cultured in 10 µg/mL cycloheximide-containing DMEM for 6 h prior to RNA isolation.

### Transcript analysis

Total RNA was extracted from cultured primary fibroblasts using the Monarch Total RNA Miniprep kit (New England BioLabs, Frankfurt, Germany). RNA concentration and purity of the samples were assessed by use of the Microplate Spectrophotometer Epoch (BioTek, Winooski, VT, USA). One microgram of total RNA was reverse transcribed (LunaScript RT Super Mix Kit, New England BioLabs). Primer sequences can be found in Supplementary Table S1. Selected PCR products were directly sequenced using the ABI BigDye Terminator Sequencing Kit (Applied Biosystems, Waltham, MA, USA) and an automated capillary sequencer (ABI 3500, Applied Biosystems). Sequence electropherograms were analyzed using Chromas v2.6.6 (Technelysium Pty Ltd, South Brisbane, Australia).

### Transcriptome sequencing data analysis

For alternative splicing analysis of *COL25A1* exons, existing transcriptome sequencing data from 42 fibroblast-derived RNA samples were used [11, 12]. For detection and quantification of individual alternative splicing events, the 2nd-pass alignment from STAR v2.7.10b [13] was used with the human Ensembl release 106 [14]. Exon-exon junctions were quantified using Leafcutter [15] and the distribution of read counts of all *COL25A1* exon-exon junctions was visualized by *ggplot2* in *R*. Sashimi plots of selected samples were visualized with the *R* package *Gviz* [16].

## Results

### Clinical reports

#### Family 1

Subjects 1 and 2 were the only children of consanguineous parents (cousins) of Egyptian descent (Table 1). During both pregnancies, decreased fetal movement was noted. Both subjects were born at 38 weeks of gestation by Caesarian section. The two siblings presented with multiple skeletal deformities, but achieved average mental and motor milestones. They were able to walk and talk as their normal peers and they attended regular school.

**Table 1:** Clinical characteristics of subjects with biallelic *COL25A1* variants.

Subject (S)	Family 1	Family 1	Family 2	Family 3	Family 4	Family 5	Family 6
	S1	S2	S3	S4	S5	S6	S7
General information							
Ethnicity	Egyptian	Egyptian	Caucasian	Saudi	Saudi	Reunion Island	Asian Indian
Sex	Male	Male	Male	NA	NA	Male	Female
Age at last examination	8 years	6 years	3 months	Fetal (18 weeks)	Fetal (13 weeks)	Fetal (25 weeks)	Fetal (13–14 weeks)
Alive/dead	Alive	Alive	Died at 3 months	Terminated pregnancy	Fetal death	Terminated pregnancy	Terminated pregnancy
*COL25A1* variant (NM_198721.4/ NP_942014.1)	Homozygous c.1730G > A; p.(Gly577Glu)	Homozygous c.1730G > A; p.(Gly577Glu)	Compound heterozygous c.367G > C; (r.300_367del; exon 3 skipping) c.1198G > T; p.(Gly400Trp)	Homozygous 4q25 (109,262,132–109,332,470) × 0 (hg38); deletion of 70,339 bp (exon 1–3 deletion)	Homozygous c.1598del; p.(Pro533Hisfs*77)	Homozygous chr4:g.108,698,908_ 108,829,130del (hg38); deletion of 130,223 bp (exon 33–38 deletion)	Homozygous c.382C > T; p.(Arg128*)
Consanguinity	Yes	Yes	No	Yes	Yes	No	No
Family history	Similarly affected younger brother (see subject 2)	Similarly affected older brother (see subject 1)	NA	Two miscarriages	1st pregnancy: hydrocephalus, short limbs, club feet, encephalocele, IUFD; 2nd pregnancy: early miscarriage; 3rd pregnancy: neural tube defect operated (20-month-old child)	Teratozoospermia (pregnancy was conceived by in vitro fertilization)	1st pregnancy: terminated because of fetal akinesia at 13 weeks of gestation; 2nd pregnancy: spontaneous first-trimester abortion
Gestational age at birth (weeks)	37 (CS)	37 (CS)	26 (CS)	NA	NA	25	NA
Length at birth in cm (*z* score)	ND	ND	37.5 (0.8 z)	NA	NA	23 (−3 z)	NA
Weight at birth in g (*z* score or SD)	2800 (−1.2 SD)	3200 (0.5 SD)	880 (−0.2 z)	NA	NA	1120 (1.5 z)	NA
OFC at birth in cm (*z* score or SD)	NA	NA	26.5 (0.8 z)	NA	NA	21 (−2 z)	NA
Current height in cm (SD)	105 (−4.1 SD)	95.5 (−3.92 SD)	NA	NA	NA	NA	NA
Current weight kg (SD)	12.2 (−4.5 SD)	12 (−3.8 SD)	NA	NA	NA	NA	NA
Current OFC in cm (SD)	50.2 (−1.52 SD)	49.1 (−1.8 SD)	NA	NA	NA	NA	NA
Age at walking	2.5 year	2 years	NA	NA	NA	NA	NA
Prenatal findings							
Complications during pregnancy	Yes; premature rupture of the membrane	No	Yes; vaginal bleeding as a result of placenta previa	US during the first trimester revealed severe IUGR and skeletal anomalies	US during the first trimester revealed increased nuchal translucency and signs of early hydrops	Anhydramnios, cystic hygroma with increased nuchal translucency	Cystic hygroma
Decreased fetal movement [HP:0001558]	Yes	Yes	No	Yes	Yes	Yes	Yes
Abnormality of the respiratory system							
Neonatal respiratory distress [HP:0002643]	No	No	Yes, respiratory failure requiring assisted ventilation	NA	NA	NA	NA
Abnormality of the musculoskeletal system							
Hypotonia [HP:0001252]	Yes	Yes	Yes, axial hypotonia	NA	NA	NA	NA
Distal amyotrophy [HP:0003693]	Yes	Yes	Yes	NA	NA	Yes	NA
Hypokinesia [HP:0002375]	Yes	Yes	Yes	NA	NA	NA	NA
Arthrogryposis multiplex congenita [HP:0002804]	Yes	Yes	Yes	The latest US showed no fetal movement (upper and lower limbs)	NA	Yes, with pterygium	Yes
Shoulder flexion contracture [HP:0003044]	Yes, with pterygium	Yes, with pterygium	Yes	ND	ND	Yes, with pterygium	ND
Elbow flexion contractures [HP:0002987]	Yes, with pterygium	Yes, with pterygium	Yes	ND	ND	Yes, with pterygium	Yes
Wrist flexion contractures [HP:0001239]	No	No	Yes	ND	ND	Yes, with pterygium	ND
Camptodactyly of finger [HP:0100490]	Yes, both hands	Yes, both hands	Yes, both hands	ND	ND	Yes, both hands	ND
Hip contracture [HP:0003273]	No	No	Yes	ND	ND	Yes, with pterygium	ND
Hip dysplasia [HP:0001385]	Yes	Yes	No	ND	ND	NA	ND
Knee flexion contractures [HP:0006380]	Yes, with pterygium (operated)	Yes, with pterygium (operated)	Yes	ND	ND	Yes, with pterygium	Yes
Rocker bottom foot [HP:0001838]	No	No	Yes, bilateral	ND	ND	ND	ND
Scoliosis [HP:0002650]	Yes	Yes	Yes	ND	ND	Yes	ND
Abnormalities of the eye							
Abnormality of eye movement [HP:0000496]	No	No	Yes, impaired abduction of the left eye	NA	NA	NA	NA
Ptosis [HP:0000508]	No	Yes, left eye	No	NA	NA	NA	NA
Miscellaneous							
Cognitive impairment [HP:0100543]	No	No	NA	NA	NA	NA	NA
Abnormal facial shape [HP:0001999]	Long and triangular face, downslanted palpebral fissures, prominent nasal bridge, pear-shaped nose, cupped and low-set ears, open mouth	Long and triangular face, downslanted palpebral fissures, prominent nasal bridge, pear-shaped nose, cupped and low-set ears, open mouth	Long and myopathic face, hypertelorism, upslanted palpebral fissures, prominent nasal bridge, low-set ears, open mouth	NA	NA	High and hairy forehead, thin lips, small chin, webbed neck	NA
Hearing abnormality [HP:0000364]	No	No	Yes	NA	NA	NA	NA
Vocal cord paralysis [HP:0001605]	No	No	NA	NA	NA	NA	NA
Other abnormalities	No	Cryptorchidism	Constipation, perforation of the ascending colon	Edema	Edema	Hydrops fetalis, fixed talipes equinovarus	Hydrops fetalis

*AMC* arthrogryposis multiplex congenital, *CS* Caesarian section, *CCDD* congenital cranial dysinnervation disorders, *IUFD* intrauterine fetal demise, *IUGR* intrauterine growth retardation, *NA* not applicable, *ND* not determined, *OFC* occipitofrontal circumference, *US* ultrasound.

**Fig. 2:**
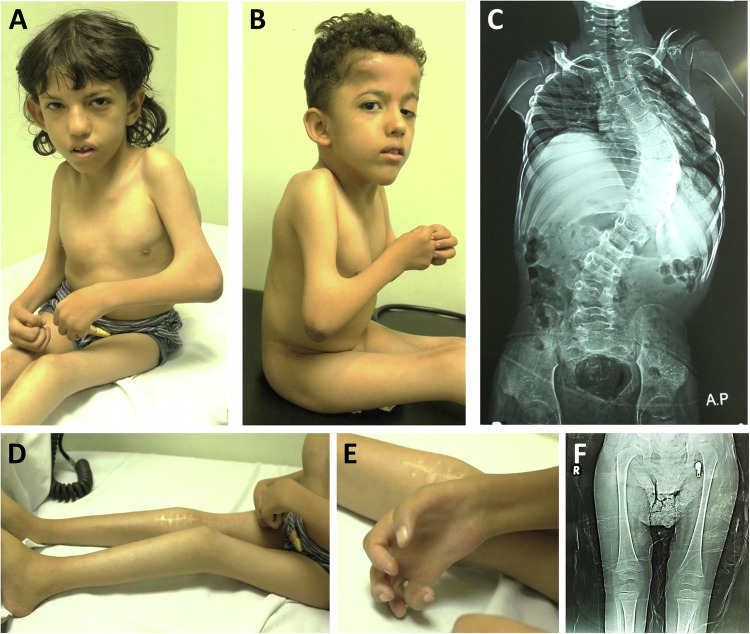
Clinical and radiological images of subject 1 at the age of 8 years and of subject 2 at the age of 6 years with the homozygous *COL25A1* c.1730G > A; p.(Gly577Glu) variant. **A**, **B** Both brothers show similar facial features and joint contractures with pterygium of multiple joints. Subject 2 has ptosis of the left eye (**B**). **C** X-ray of the spine shows S-shaped scoliosis in subject 1. **D** Subject 1 shows limited extension of the left knee and a scar on the right knee from previous surgery to correct arthrogryposis. **E** The right hand of subject 1 shows camptodactyly and faded dermatoglyphics with small thenar and hypothenar eminences. **F** X-ray of lower limbs reveals flattened knee epiphyses and mild metaphyseal widening in the femur and tibia of subject 1.

On examination at the age of 8 years, subject 1 had a weight of 12.2 kg (−4.5 SD), a height of 105 cm (−4.1 SD), and an occipitofrontal head circumference (OFC) of 50.2 cm (−1.52 SD). Subject 2 was 6 years old and weighed 12 kg (−3.8 SD), had a height of 95.5 cm (−3.92 SD), and an OFC of 49.1 cm (−1.8 SD). The two brothers had almost the same facial features such as a triangular face with a pointed chin, cupped ears, pear-shaped nose, short philtrum, tented upper lip, and webbed short neck (Fig. 2). The younger brother had left-sided ptosis. The two siblings showed contractures of the large and small joints. They had limited movement of shoulders with axillary folds, bilateral contractures of elbows with pterygium, limited knee movement, camptodactyly, bilateral adducted thumbs, bilateral single transverse palmar crease with faded dermatoglyphics, and overlapping toes. Both had flared lower ribs and kyphoscoliosis (Fig. 2). Neurological examination revealed normal muscle tone, but reflexes were difficult to elicit. Subject 2 had a history of bilateral undescended testes.

Karyotyping, brain imaging, auditory brainstem response test, fundus examination, electromyography, nerve conduction velocity, and other laboratory tests were unremarkable. Skeletal surveys showed more pronounced scoliosis in the older brother and less affection in the younger. Both had mild flattened epiphyses and mild metaphyseal widening of long bones (Fig. 2).

#### Family 2

Subject 3 was the first child of a healthy non-consanguineous Caucasian couple (Table 1). The family history was remarkable for Graves’ disease in the mother, scoliosis in the father and paternal grandmother, trisomy 13 in two of the father’s cousins, and sudden cardiac death in the maternal family. The pregnancy was complicated by recurrent vaginal bleeding caused by placenta previa. At 25 weeks’ gestation, the mother experienced premature contractions and rupture of membranes, resulting in the premature birth of the boy by Caesarian section at 26 weeks’ gestation. His birth measurements were normal, with weight of 880 g (−0.2 z), length of 37.5 cm (0.8 z), and OFC of 26.5 cm (0.8 z). He had delayed adaptation with Apgar scores of 5/7/8 and respiratory failure requiring assisted ventilation and admission to the neonatal intensive care unit. Examination at birth revealed decreased spontaneous movements, limited shoulder movement, flexion contracture of elbows, camptodactyly of fingers and overlapping fingers, knee contracture with restrictions in flexion of the knee joint, bilateral talipes equinovarus, and rocker bottom feet. He had hypoplasia involving the skeletal musculature of arms and legs, a long myopathic face, hypertelorism, upslanted palpebral fissures, low-set ears, a high nasal bridge, and an open mouth. He exhibited poor sucking and swallowing, resulting in feeding difficulties and the need for gastrostomy tube feeding.

A thoracic spine X-ray showed a severe scoliosis. Otoacoustic emissions testing revealed abnormal results, however, an additional testing was not performed. Ultrasound of the brain, heart, and abdomen as well as ophthalmologic examination were normal, except for preterm anomalies such as persistent patent ductus venosus. Karyotyping revealed a normal male karyotype (46,XY).

He had constipation from day 1. At 4 weeks of age, he developed a mechanical ileus and perforation of the ascending colon. A double-barrel ileostomy was then formed, allowing for parenteral nutrition.

On examination at the age of 3 months, he showed axial hypotonia and diminished spontaneous movements. The boy was able to open his eyes, however, a detailed ophthalmological examination was not possible. The parents reported that his gaze could fix on objects and track them, although the left eye showed impaired abduction. Contractures did not substantially improve over time and assisted ventilation was still required. He died at age 3 months and 9 days in a palliative care setting. A postmortem muscle biopsy revealed minor signs of an unspecified myopathy.

#### Family 3

Subject 4 was the fetus of a 27-year-old female with thyroid cancer, post-total thyroidectomy, and radioactive iodine therapy (Table 1). Family history was remarkable for two miscarriages. Examination at 13 weeks of gestation raised some concerns with crown-rump length below the fifth centile and large nuchal translucency of 4.5 mm. Ultrasound at 18 weeks of gestation revealed bilateral short humerus, femur, and tibiae. The fetus had a cloverleaf skull shape. The pregnancy was terminated.

#### Family 4

Subject 5 was the fetus of a healthy first-degree consanguineous Arab couple (Table 1). There was a positive family history with the first pregnancy with hydrocephalus, short limbs, club feet, encephalocele, and intrauterine fetal demise, the second pregnancy was an early miscarriage, and the third child with neural tube defect survived after surgery. Examination at 13 weeks of gestation showed increased nuchal translucency and signs of early hydrops. Ultrasound revealed upper- and lower-limb joint contractures that were followed by intrauterine fetal demise 3 weeks later.

#### Family 5

**Fig. 3:**
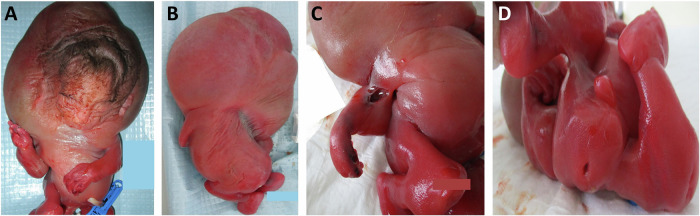
Photographs of subject 6 taken at autopsy following pregnancy termination at 25 weeks’ gestation. **A** Full body front view of the fetus showing hydrops with profound edema of the face, head, and neck. The fetus had a small chin, thin lips, and a high, hairy forehead. **B** Full body back view showing the large head edema and curvature of the spine. **C**, **D** The fetus showed arthrogryposis with multiple pterygia and edema affecting all limbs.

Subject 6 was a fetus who presented with cystic hygroma and increased nuchal translucency of 15.6 mm in the first trimester (Table 1). Follow-up ultrasound revealed decreased fetal movements, hydrops fetalis, and fixed talipes equinovarus. The pregnancy was terminated at 25 weeks’ gestation. Autopsy of the fetus showed hydrops fetalis with profound edema of the face and head, multiple pterygia of all joints (Fig. 3), small thorax, and generalized muscle hypoplasia.

#### Family 6

Subject 7 was the fetus (third pregnancy) of a healthy non-consanguineous Asian Indian couple (Table 1). The 29-year-old female presented for evaluation at ~13 weeks’ gestation. The first pregnancy was terminated because of fetal akinesia and the second pregnancy ended in spontaneous abortion in the first trimester. At 13 weeks and 2 days’ gestation, real-time B-mode ultrasound showed generalized edema, consistent with non-immune fetal hydrops. Fetal assessment revealed the absence of movement. Bilateral lymphatic cysts in the neck, consistent with cystic hygroma, and mild bilateral pleural effusion were noted. Both upper limbs were persistently flexed at the elbow joints and both lower limbs were flexed at the knee joints, suggesting arthrogryposis. The pregnancy was terminated at 13–14 weeks.

### Genetic findings

All identified *COL25A1* variants are summarized in Table 1 and shown in Fig. 1. In subjects 1 and 2, two similarly AMC-affected brothers, single ES followed by segregation analysis revealed the homozygous *COL25A1* missense variant NM_198721.4:c.1730G > A; p.(Gly577Glu) in both. In subject 3, who had a severe AMC form and deceased at the age of 3 months, trio ES detected the compound heterozygous *COL25A1* variants c.367G > C and c.1198G > T, predicting the amino acid substitution p.(Gly123Arg) and p.(Gly400Trp), respectively (Supplementary Fig. S1A). In subjects 4 to 7, four unrelated fetuses with severe and complex phenotypes, distinct homozygous *COL25A1* loss-of-function variants were identified. A homozygous 70,339-bp deletion encompassing the first three exons of *COL25A1* and *COL25A1-DT*, a long non-coding RNA gene, was found in subject 4 [4q25(109,262,132–109,332,470)x0; hg38] by chromosomal microarray analysis. In subject 5, the homozygous 1-bp deletion c.1598del; p.(Pro533Hisfs*77) was detected by trio ES. Both *COL25A1* variants and a brief phenotypic note for subjects 4 and 5 have been previously reported (families F6581 and F6582 in [17]). In subject 6, trio GS revealed a homozygous deletion of 130,223 bp in 4q25 [chr4(hg38):g.108,698,908–108,829,130del], encompassing the last six exons (33–38) of the longest *COL25A1* transcript variant and the last three and two exons in transcript variants NM_032518.4 and NM_001256074.3, respectively. *ETNPPL* and the pseudogene *RCC2P8* were also deleted (Supplementary Fig. S2). Single ES in subject 7 identified the homozygous nonsense variant c.382C > T; p.(Arg128*) (Supplementary Fig. S1B). Healthy parents of all seven subjects were heterozygous carriers of a *COL25A1* variant (Supplementary Material and Methods and Supplementary Figs. S1 and S2).

All *COL25A1* variants were absent from population databases, with the exception of the c.382C > T; p.(Arg128*) variant, which had an allele frequency of about 0.0007% in the gnomAD database v4.1.0 [18] and in the Regeneron Genetics Center Million Exome data [19]. The three missense variants c.367G > C; p.(Gly123Arg), c.1198G > T; p.(Gly400Trp), and c.1730G > A; p.(Gly577Glu) were predicted to be pathogenic by the in silico programs AlphaMissense, CADD, and REVEL (Supplementary Table S2). The G-to-C change at position c.367 affects the last nucleotide of exon 3 and the c.1198G > T variant the first nucleotide of exon 23. For the c.367G > C change, all five splice prediction programs predicted an effect on splicing, in particular the loss of the splice donor site in intron 3. In contrast, the in silico analyses provided no evidence for the variant c.1198G > T to impact *COL25A1* pre-mRNA splicing (Supplementary Tables S2 and S3). Importantly, the canonical splice acceptor site in intron 22 was not detected by any of the used splice prediction programs (Supplementary Table S3), indicating a weak splice acceptor site. Following the guidelines of the American College of Medical Genetics and Genomics and the Association for Molecular Pathology [20], we interpreted all *COL25A1* variants identified here and those previously reported. Except for the homozygous missense variant c.1450A > G; p.(Lys484Glu) described in a patient with AMC [4], that we classified as a variant of uncertain significance, all other 13 variants were interpreted as likely pathogenic or pathogenic variants (Supplementary Table S2).

### *COL25A1* transcript analysis in subject 3 fibroblasts

**Fig. 4:**
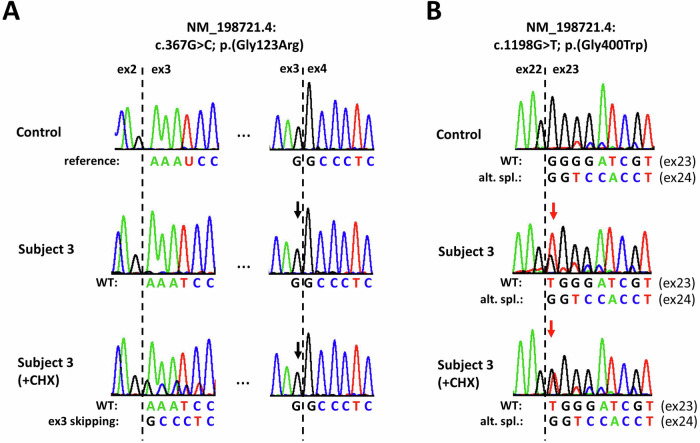
*COL25A1* transcript analysis in fibroblasts from subject 3 and a control. Partial sequence electropherograms after direct Sanger sequencing of RT-PCR fragments obtained with a forward primer in exon 2 and a reverse primer in exon 4 (**A**) or a forward primer in exon 20 and a reverse primer in exon 24 (**B**) using cDNA from fibroblasts of a control (upper panel) and subject 3 (middle and lower panels). Subject 3’s fibroblasts were either untreated (middle panel) or treated with the NMD inhibitor cycloheximide (+CHX; lower panel) before RNA isolation. Exon numbering is given. Exon-exon junctions are marked by dotted lines. **A** Sequence traces show canonically spliced *COL25A1* transcripts in control cells (upper panel). In untreated subject 3-derived fibroblasts only *COL25A1* transcripts expressed from the maternal allele were identified as the reference guanine at position r.367 was detected (black arrow; middle panel). CHX treatment of subject 3 cells revealed transcripts with exon 2 directly spliced to exon 4 in *COL25A1* transcripts (exon 3 skipping) in addition to canonically spliced transcripts (lower panel). Of note, the sequencing trace showing *COL25A1* transcripts with exon 3 skipping (lower panel) are 70 bp shorter than those of canonically spliced *COL25A1* transcripts, which explains why only peaks representing *COL25A1* transcripts expressed from the maternal allele (with r.367g) are observed at the exon 3-exon 4 junction. **B** Partial sequence electropherograms show canonical splicing of exon 22 to exon 23 in control cells. In addition, a second sequence representing alternatively spliced *COL25A1* transcripts with an exon 22-exon 24 junction is superimposed on the reference sequence (upper panel). In untreated and CHX-treated fibroblasts from subject 3, sequence traces of canonically spliced *COL25A1* transcripts with the r.1198g > u variant (thymine marked by a red arrow) are observed in addition to alternatively spliced transcripts with skipping of exon 23 (superimposed on the reference sequence in middle and lower panels). alt. spl. alternatively spliced, ex exon, NMD nonsense-mediated mRNA decay.

We investigated the effect of the paternally inherited variant c.367G > C (in exon 3) and the maternally inherited variant c.1198G > T (in exon 23) on *COL25A1* pre-mRNA splicing using RNA isolated from skin fibroblasts of subject 3 and a control. We used a forward primer in exon 2 and a reverse primer in exon 4 in RT-PCRs (Supplementary Table S1) and generated an amplicon of the expected wild-type size in patient and control cells. Sanger sequencing of the subject 3-derived amplicon revealed only *COL25A1* transcripts with the wild-type base guanine at position c.367 (Fig. 4A), suggesting expression of *COL25A1* mRNAs only from the maternal allele (with the c.1198G > T variant) in subject 3 fibroblasts. As the c.367G > C variant was predicted to cause loss of the splice donor site in intron 3 (Supplementary Table S3), this could lead to exon 3 skipping, followed by a shift in the reading frame, introduction of a premature stop codon, and nonsense-mediated mRNA decay (NMD) of aberrantly spliced *COL25A1* transcripts. To inhibit NMD, we treated subject 3- and control-derived fibroblasts with cycloheximide (CHX), isolated RNA, and repeated the *COL25A1* RT-PCR. We generated a single product with the expected size from cDNA of control cells, while we obtained an RT-PCR product with the expected size in addition to a smaller amplicon from cDNA of subject 3 fibroblasts. Sequencing of subject 3-derived amplicons revealed the canonically spliced exon 2-exon 3 junction in one sequence. In the other sequence superimposed on the reference sequence, exon 2 was spliced directly to exon 4 (Fig. 4A). The data demonstrate that the c.367G > C variant causes skipping of exon 3 in *COL25A1* mRNAs, leading to a frameshift and a premature stop codon [r.300_367del; p.(Ser101Profs*7)]. These aberrantly spliced *COL25A1* transcripts were only detected in CHX-treated subject 3 fibroblasts, suggesting their efficient clearance by NMD.

To analyze possible aberrant splicing due to the maternally inherited variant c.1198G > T (in exon 23), we used a forward primer in exon 20 and a reverse primer in exon 24 and amplified two distinct RT-PCR products using cDNA from fibroblasts of subject 3 and controls. Sanger sequencing revealed canonical splicing of exon 22 to exon 23 in the *COL25A1* mRNA as identified in one sequence trace. The second sequence that was superimposed on the reference sequence showed exon 22 spliced to exon 24 in both subject 3 and control sequences (Fig. 4B). After CHX treatment of subject 3 cells, the same RT-PCR amplicons and sequences were generated as in untreated cells (Fig. 4B). Our data show that the *COL25A1* variant c.1198G > T does not cause aberrant splicing of *COL25A1* pre-mRNAs. Interestingly, we identified alternative splicing of exon 23 in human fibroblasts, likely because of the weak splice acceptor site in intron 22 (Supplementary Table S3), leading to the expression of *COL25A1* transcript variants with and without this *in-frame* exon.

### Alternative splicing of *COL25A1* exons in primary human fibroblasts

Our finding that exon 23 of *COL25A1* is alternatively spliced in fibroblasts and literature data demonstrating that certain exons in genes encoding various collagens are alternatively spliced [21–23], led us to analyze alternative splicing of *COL25A1* exons in existing transcriptome sequencing data from 42 primary human skin fibroblasts from individuals without *COL25A1* pathogenic variants [11, 12]. We evaluated transcriptome data for alternative splicing events of single and multiple exons. In Supplementary Fig. S3, we show all identified *COL25A1* exon-exon junctions. According to the MANE Select transcript NM_198721.4, we found known alternative splicing events, such as skipping of exon 22 and exon 25 (Supplementary Fig. S3E, F). Furthermore, we detected several novel exon-exon junctions and identified alternative splicing of the single *in-frame* exons 6, 9, 16, 19, 23, 32, 34, and 36 (Fig. 1B and Supplementary Fig. S3B–E, G) as well as alternative splicing of multiple subsequent *in-frame* exons, such as exons 9-11, exons 22-23, exons 22-24, and exons 33-34 (Fig. 1B and Supplementary Fig. S3C, E, G). The predominant transcript variant expressed in fibroblasts was NM_001256074.3 (ENST00000399127.5) (Fig. 1B). Together, the data show complex alternative splicing of multiple *COL25A1* exons in fibroblasts, suggesting that different collagen XXV isoforms can be produced from these mRNAs.

## Discussion

Here, we report on seven subjects from six families with biallelic *COL25A1* variants, showing clinical features of AMC in six individuals of varying severity. The phenotype of subjects 1 and 2, two brothers, was at the milder end of the spectrum as they had flexion contractures of multiple joints and scoliosis. Subject 3 was more severely affected than the siblings. He showed akinesia at birth, flexion contractures of limbs, muscular hypoplasia, hypotonia, and feeding difficulties. He died of respiratory failure as an infant. Eye abnormalities were present in subject 2 who had left-sided ptosis, but no abnormality of eye movement, while subject 3 had impaired abduction of the left eye. The clinical features in subjects 1–3 were similar to those reported previously in five patients with biallelic *COL25A1* variants and AMC [4]. Main clinical findings were contractures with or without CCDD. Three patients had respiratory muscle involvement. The common denominator of AMC is decreased or absent fetal movement (fetal hypo- or akinesia) [5] that was present in subjects 1–3 described here and in two of the five previously reported patients [4]. Subjects 4–7, the four fetuses described here, had a lethal condition or the pregnancy was terminated because of severe abnormalities. Subject 4 showed growth restriction with short extremities and a cloverleaf skull. The other three fetuses had arthrogryposis with or without multiple pterygia of the limbs and edema (hydrops). Fetal arthrogryposis associated with nuchal edema or hydrops, pterygia, and/or growth restriction is often indicative of a lethal condition [24]. However, given the limited number of reported cases, phenotypic categorization remains challenging. With these findings, we expand the phenotypic spectrum and show that biallelic *COL25A1* variants can cause a fetal lethal condition.

Col25a1 is important for intramuscular motor innervation and development of cranial motor neurons [7]. It induces intramuscular axon growth by interacting with the receptor protein tyrosine phosphatases σ and δ (PTPσ/δ) on motor axons [10]. Several other proteins, such as ECEL1, are involved in the peripheral axon branching process. ECEL1 is required for axon arborization of spinal motor nerves and the formation of the neuromuscular junction [25, 26]. Biallelic *ECEL1* variants cause distal arthrogryposis, type 5D (MIM 615065) [27]. The associated clinical spectrum is similar to that caused by *COL25A1* variants. Patients have congenital contractures affecting mostly the distal joints of upper and lower limbs, club feet, hip dislocation, short stature, and scoliosis. Ocular findings, such as ptosis, severe ophthalmoplegia, and astigmatism, are common. At the most severe end of the spectrum, fetuses with multiple pterygia and arthrogryposis have been described [28–30]. Mice deficient of Ecel1 or carrying specific *Ecel1* pathogenic missense variants show abnormal axonal branching of motor nerve and neuromuscular junction formation, suggesting that motor innervation defects underlie the ECEL1-related congenital contracture disorders [31, 32]. We assume that reduced or absent intramuscular motor innervation may also underlie the variable AMC phenotypes with or without CCDD in individuals with biallelic *COL25A1* variants. In line with this, CCDD-associated COL25A1 patient variant proteins show reduced binding to PTPσ/δ and attenuate axon attraction [10]. In addition, mice with *Col25a1* knockout and muscle-specific deletion of *Col25a1* show a failure of motor axon entry into targeted muscles, followed by apoptosis of spinal motor neurons [9, 10].

The disease-associated *COL25A1* variants reported by others and here comprise three microdeletions of ~70, ~124, and ~130 kb encompassing exons 1–3, exons 4–11, and exons 33–38. The exon 4–11 deletion [chr4:109,852,901–109,976,457 (UCSC GRCh37/hg19); chr4:108,931,745–109,055,301 (GRCh38/hg38)] has been reported to have a size of 12.4 kb [3], however, according to the provided chromosomal position, the deletion has a size of 123,556 bp (~124 kb). The deletion encompassing *COL25A1* exons 33–38 also comprises a pseudogene and *ETNPPL*, which has not yet been associated with a human disorder (MIM 614682). *ETNPPL* encodes the ethanolamine phosphate phospholyase, an enzyme implicated in the Kennedy pathway of phosphatidylethanolamine biosynthesis. In mice, Etnppl regulates plasma lipoprotein metabolism [33]. The data suggest that the homozygous loss of *ETNPPL* did not modify or contribute to the severe phenotype in the fetus (subject 6). In addition to the microdeletions, two nonsense, one frameshift, two splice site variants, one variant impacting splicing, and five missense variants that are predicted to cause amino acid substitutions are known up to date (this report and [3, 4]). Four of the five amino acid substitutions change a glycine for another amino acid residue that are most common pathogenic missense variants in genes encoding collagens [34]. A genotype-phenotype correlation does not seem to exist, especially as one individual with isolated CCDD was compound heterozygous for two apparently *COL25A1* null alleles, such as the nonsense variant c.1489G > T; p.(Gly497*) in exon 28 and the intragenic ~124-kb deletion [2, 3]. Similarly, the four severely affected fetuses reported here carried homozygous null alleles. The other patients with biallelic splice site and missense variants had isolated CCDD or AMC with or without CCDD (this work and [3, 4]), suggesting that these types of variants are associated with intermediate phenotypes.

Our bioinformatics analysis of *COL25A1* mRNAs expressed in fibroblasts (without *COL25A1* pathogenic variants) revealed alternative splicing of 13 novel *in-frame* exons in addition to the known alternatively spliced exons (Fig. 1B). The 13 exons encode part of the NC2, COL1, COL2 or COL3 domain (Fig. 1A). For the paralogous gene *COL13A1*, encoding the MACIT-type collagen XIII, extensive tissue-specific alternative splicing of ten exons affecting the NC2, COL1, and COL3 units has been reported. A minimum of 17 *COL13A1* transcript variants exist [21]. As the single MACIT *col-99* gene in *Caenorhabditis elegans* also undergoes alternative splicing [35], complex combinations of exons on the RNA level seem to be common to MACIT collagen genes and important for their molecular function. The presence of pathogenic variants in alternatively spliced exons of a disease gene can result in phenotypes that are distinct from those caused by variants in constitutively spliced exons. This is because pathogenic variants in alternatively spliced exons are absent in mRNAs expressed in certain disease-relevant tissues, often leading to a milder phenotype [34, 36]. For example, a heterozygous pathogenic variant in the alternatively spliced exon 9 of *COL11A1* in trans with another *COL11A1* pathogenic variant results in Stickler syndrome and not in the often lethal fibrochondrogenesis [37]. As this phenomenon is well known for genes encoding collagens [34], we hypothesize that naturally occurring alternative splicing of certain *COL25A1* exons results in the elimination of an exon with a loss-of-function variant in mRNAs expressed in certain tissues followed by the production of a functional collagen XXV isoform. For example, the exclusion of exon 28 with the c.1489G > T; p.(Gly497*) variant in *COL25A1* mRNAs expressed in various tissues may lead to the synthesis of functionally intact COL25A1 isoforms, while in cranial motor neurons, exon 28 with the pathogenic variant could be highly included in mature *COL25A1* transcripts. Such a scenario would explain why the nonsense variant in trans with the multiple-exon deletion caused isolated CCDD and not a severe fetal phenotype (Fig. 1C) [2, 3]. In line with these assumptions, analysis of human transcriptome sequencing data across tissues and individuals showed interindividual variation in the expression and splicing of many genes that may contribute to individual phenotypes [38, 39].

The homozygous *COL25A1* missense variant c.1144G > A; p.(Gly382Arg) has been associated with ophthalmic phenotypes and is located in the constitutively spliced exon 21 (Fig. 1B, C). Similar to exon 28 with the variant p.(Gly497*), also exon 21 could be alternatively spliced in various tissues and could be present in *COL25A1* mRNAs expressed in cranial motor neurons, leading to CCDD. Functional studies of the *COL25A1* variant p.(Gly382Arg) showed less stability of the ectopically expressed mutant protein compared to the wildtype, but only at higher temperatures. Complex formation of COL25A1^Gly382Arg^ was reduced with PTPσ but was similar with sAPP and NTN1 compared to COL25A1 wildtype [3, 10]. These data suggest that the *COL25A1* p.(Gly382Arg) variant is hypomorphic, likely leaving some function of the protein intact, and may be associated with a mild (ocular) phenotype.

With the limited data available, attempts at genotype-phenotype correlation for biallelic *COL25A1* variants are difficult. Studies with more extensive transcriptomic data across different tissues and developmental stages in humans would be required to identify alternatively and constitutively spliced *COL25A1* exons in different tissues and organs and possibly also isoform switching from prenatal to postnatal stage.

## Supplementary information

Supplementary Information

## Data Availability

The data that support the findings of this study are available within the paper and in the supplementary information. Chromosomal microarray and genome and exome sequencing data are not publicly available due to privacy or ethical restrictions. The novel *COL25A1* variants reported in subjects 1–7 in this manuscript were submitted to the LOVD database (https://databases.lovd.nl/shared/genes/COL25A1), with the LOVD variant IDs #0001012735, #0001012736, #0001012806, #0001012807, #0001012808, #0001012809, #0001012923, and #0001012924.
