# Supplementary material for: Novel biallelic COL25A1 variants broaden the clinical spectrum from congenital cranial dysinnervation disorders to fetal lethal phenotypes
Source: Eur J Hum Genet. 2025 Mar 29;33(12):1616–26. doi: 10.1038/s41431-025-01839-4 (PMC12669723; doi:10.1038/s41431-025-01839-4)
Supplement: Supplementary file 1 — Supplementary Information [file 41431_2025_1839_MOESM1_ESM.docx]

**SUPPLEMENTARY INFORMATION**

**Novel biallelic *COL25A1* variants broaden the clinical spectrum from congenital cranial dysinnervation disorders to fetal lethal phenotypes**

Frederike L. Harms^1^, Christian Müller^2^, Fanny Kortüm^1^, Maja Hempel^1,3^, Malik Alawi^2^, Maha S. Zaki^4^, Rasha M. Elhossini^4^, Mohamed S. Abdel-Hamid^5^, Lama AlAbdi^6^, Fowzan S. Alkuraya^6^, Wesam Kurdi^7^, Tristan Celse^8^, Marta Spodenkiewicz^8^, Tiphany Laurens^8^, Klaus Dieterich^9,10^, Sujatha Jagadeesh^11^, Sandesh Salvankar^12^, Katta M. Girisha^12,13,14^, Kerstin Kutsche^1,15^

^1^ Institute of Human Genetics, University Medical Center Hamburg-Eppendorf, Hamburg, Germany

^2^ Bioinformatics Core, University Medical Center Hamburg-Eppendorf, Hamburg, Germany

^3^ Present address: Institute of Human Genetics, Heidelberg University, Heidelberg, Germany

^4^ Clinical Genetics Department, Human Genetics and Genome Research Institute, National Research Centre, Cairo, Egypt

^5^ Medical Molecular Genetics Department, Human Genetics and Genome Research Institute, National Research Centre, Cairo, Egypt

^6^ Department of Translational Genomics, Center for Genomic Medicine, King Faisal Specialist Hospital and Research Center, Riyadh, Saudi Arabia

^7^ Department of Obstetrics and Gynecology, King Faisal Specialist Hospital and Research Center, Riyadh, Saudi Arabia

^8^ Service de Génétique Médicale, Centre Hospitalier Universitaire de La Réunion, La Réunion, France.

^9^ Universite Grenoble Alpes, Inserm, U1209, CHU Grenoble Alpes, France

^10^ Medical Genetics, Institute of Advanced Biosciences, 38000 Grenoble, France

^11^ Mediscan Systems, Chennai, India

^12^ Suma Genomics Private Limited, Manipal, India

^13^ Department of Medical Genetics, Kasturba Medical College, Manipal, Manipal Academy of Higher Education, Manipal, India

^14^ Department of Genetics, College of Medicine and Health Sciences, Sultan Qaboos University, Muscat, Oman

^15^ German Center for Child and Adolescent Health (DZKJ), partner site Hamburg, Hamburg, Germany

Correspondence to:

Kerstin Kutsche, PhD

Institute of Human Genetics

University Medical Center Hamburg-Eppendorf

Martinistraße 52

20246 Hamburg

Germany

email: kkutsche@uke.de

**SUPPLEMENTARY MATERIALS AND METHODS**

**Genetic analyses**

Subjects 1 and 2

After obtaining a signed informed consent and the approval of the Medical Research Ethics Committee of the National Research Centre, Cairo, Egypt (Approval number: 20066), genomic DNA was extracted from peripheral blood samples of subjects 1 and 2 and their parents using Qiagen Blood DNA Kit (Qiagen, Hilden, Germany) and quantified by a Nanodrop 2000 system (Thermo Fisher Scientific, Waltham, MA, USA). Single whole exome sequencing was performed in a research unit for subject 1 using the SureSelect Human All Exome 50 Mb Kit (Agilent, Santa Clara, CA, USA) and the Illumina NovaSeq 6000 sequencer (Illumina, San Diego, CA, USA). The obtained sequences were aligned to UCSC human genome GRCh37/hg19 and variants were verified through the GATK pipeline. Annotation of variants was done using BaseSpace Variant Interpreter Server. Identified variants were checked against public genetic databases like Genome Aggregation Database (gnomAD, https://gnomad.broadinstitute.org/), 1000 Genomes (www.1000genomes.org), and dbSNP (http://www.ncbi.nlm.nih.gov/SNP/). Pathogenicity of detected missense and splice site variants was predicted using various bioinformatics tools as SIFT (https://provean.jcvi.org/protein), PolyPhen-2 (https://genetics.bwh.harvard.edu/pph2/) and MutationTaster (https://www.mutationtaster.org/). Only rare variants (novel or with an allele frequency ≤ 0.001 in gnomAD) related to the patients’ phenotype were selected.

Sanger sequencing was used for confirmation and segregation of the *COL25A1* variant c.1730G>A; p.(Gly577Glu) in subject 2 and parents. Exon 33 harboring this variant was amplified by standard PCR. Primer sequences can be found in **Supplementary Table S1**. PCR products were purified using the Exo-SAP PCR Clean-up kit (Fermentas, Germany), sequenced in both directions using the BigDye Terminator v3.1 Cycle Sequencing Kit (Applied Biosystems, Foster City, CA, USA), and analyzed on the ABI Prism 3500 Genetic Analyzer (Applied Biosystems) according to manufacturer's instructions.

Subject 3

Genomic DNA from subject 3 and parents was extracted from peripheral blood samples using standard procedures. Coding DNA fragments were enriched with the SureSelect Human All Exon V6 Kit (Agilent) and captured libraries were then loaded and sequenced on the HiSeq platform (Illumina). Reads were aligned to the human reference genome (UCSC GRCh37/hg19) using the Burrows-Wheeler Aligner (BWA mem, v0.7.17-r1188) [1] and genetic variations were detected using the Genome Analysis Toolkit (GATK, v3.8) [2]. We annotated single nucleotide variants and insertions/deletions using AnnoVar (v2018-04-16) [3]. *De novo* and biallelic exonic and intronic variants located at exon-intron boundaries ranging from -10 to +10 that were private (absent in the gnomAD database v2.1.1, v3.1.2, and v4.1.0) or rare (with a minor allele frequency [MAF] ≤ 0.1% and no homozygotes in the gnomAD database v2.1.1, v3.1.2, and v4.1.0) were retained. Variants with poor depth of sequencing coverage (total read depth <10) and in low quality regions (checked in IGV) were discarded. Sanger sequencing was used for confirmation of the *COL25A1* variants in subject 3 and parents (**Supplementary Table S1 and Supplementary Fig. S1A**).

Subject 4

Chromosomal microarray analysis was performed using a sample from subject 4 and ran on Cytoscan HD array (Affymetrix, Santa Clara, CA, USA). Results were analyzed using chromosomal analysis suite (ChAS, Affymetrix). Carrier status was confirmed for the parents using qPCR.

Subject 5

Trio exome sequencing was performed using DNA from subject 5 and parents. The samples were sequenced using the NovaSeq 6000 sequencer (Illumina). Sequences were mapped to and analyzed in comparison with the human genome build Genome Reference (hg19). Upon variant identification, segregation analysis was performed using samples from all available family members using Sanger sequencing.

Subject 6

For subject 6 and parents, genome sequencing was performed following the recommendations of the France Genomic Medicine Plan. Genomic DNA extracted from whole blood was sequenced according to standard procedures for a PCR-free genome on a NovaSeq 6000 sequencer (Illumina). Sequencing data were aligned to the GRCh38p13 full assembly using BWA v0.7. Variants were called by several algorithms including GATK4, Bcftools v1.10, Manta v1.6, CNVnator v0.4, and annotated using the variant effect predictor. Detected variants were prioritized using in-house procedures. Further details are available on request on http://www.auragen.fr.

Subject 7

Exome sequencing was performed using the Twist Exome 2.0 Capture Kit (TWIST Bioscience, South San Francisco, CA, USA). Captured libraries were loaded and sequenced on the NovaSeq 6000 sequencer (Illumina). Analysis was performed using Franklin by Genoox (Palo Alto, CA, USA) against the population databases and in-house data. Sanger sequencing was used for confirmation and segregation of the *COL25A1* variant in subject 7 and parents (**Supplementary Table S1 and Supplementary Fig. S1B**).

**SUPPLEMENTARY FIGURES**

**
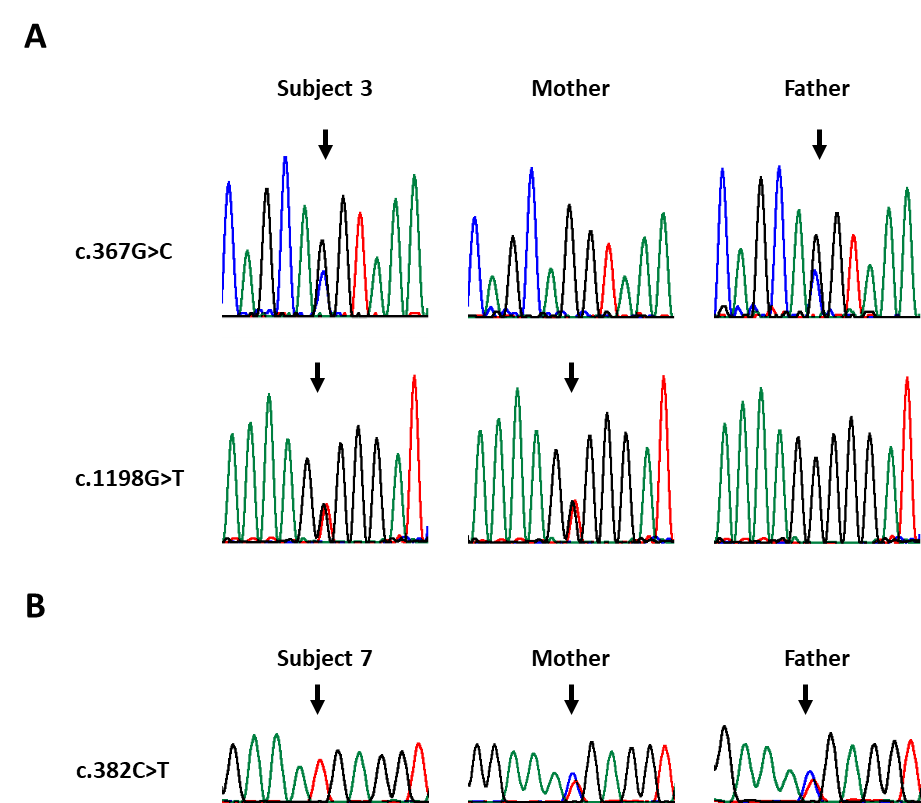
**

**Supplementary Fig. S1. Validation and/or segregation of the *COL25A1* variants in subjects 3 and 7 and their parents by Sanger sequencing. (A)** Partial sequence electropherograms show the compound heterozygous *COL25A1* variants c.367G>C and c.1198G>T in DNA isolated from leukocytes of subject 3. The heterozygous *COL25A1* variant c.367G>C was present in leukocyte-derived DNA of the healthy father and the heterozygous variant c.1198G>T in leukocyte-derived DNA of the healthy mother. **(B)** Partial sequence electropherograms show the homozygous *COL25A1* variant c.382C>T in DNA isolated from leukocytes of subject 7. The variant c.382C>T was present in leukocyte-derived DNA of the mother and father in the heterozygous state. Variants are given according to *COL25A1* reference sequence NM_198721.4. Arrows point to the position of the variants.

**
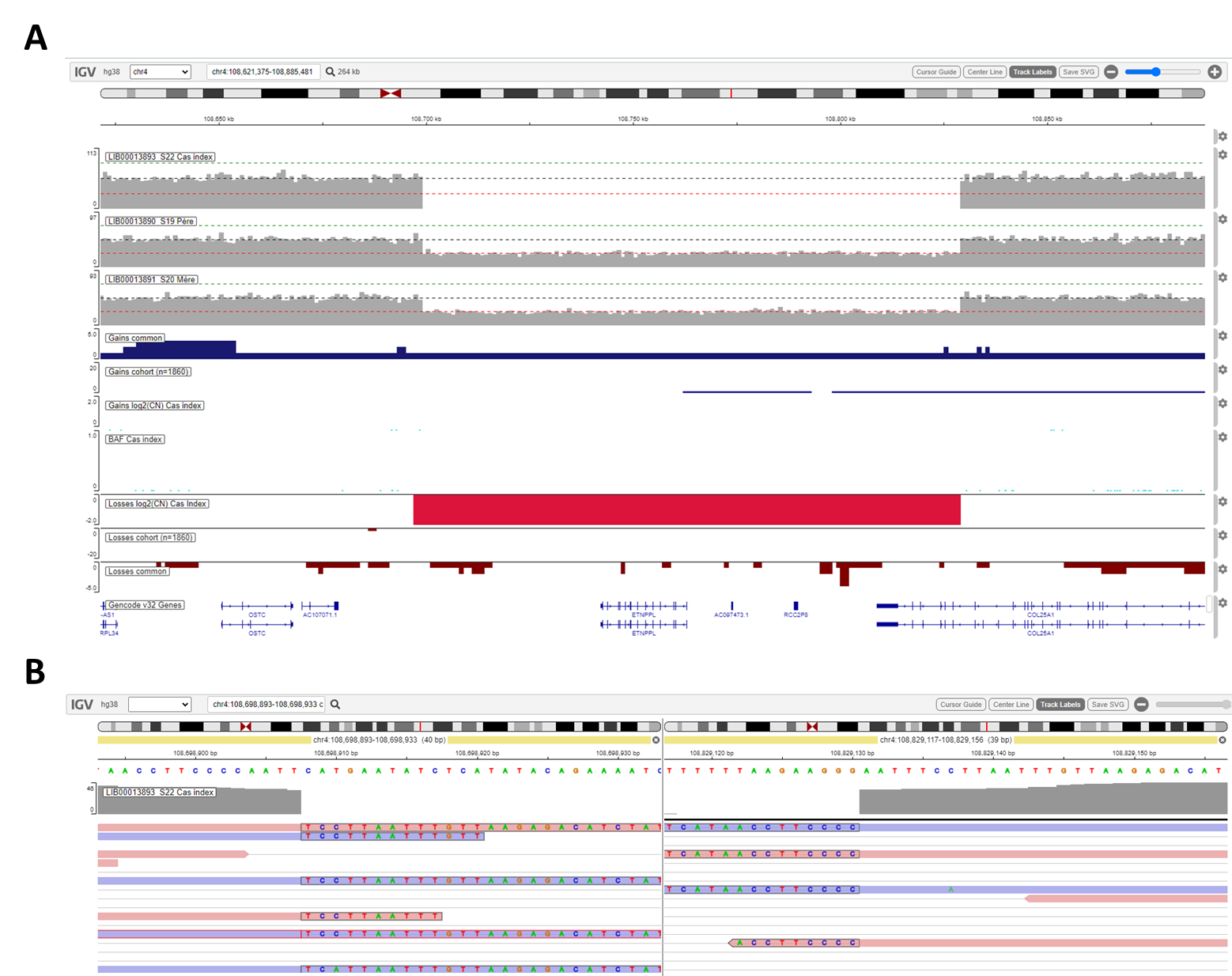
**

**Supplementary Fig. S2. Integrative Genomics Viewer (IGV) visualizations of the homozygous 130-kb deletion encompassing the 3’ end of *COL25A1* in subject 6. (A)** IGV visualization of the 4q25 region showing the homozygous deletion (red bar) in subject 6 (chr4[hg38]:g.108,698,908_108,829,130del) inherited from both heterozygous parents and encompassing *ETNPPL*, *RCC2P8*, and part of *COL25A1* (exons 33 to 38). **(B)** IGV visualization of individual sequence reads spanning the deletion breakpoints.

*
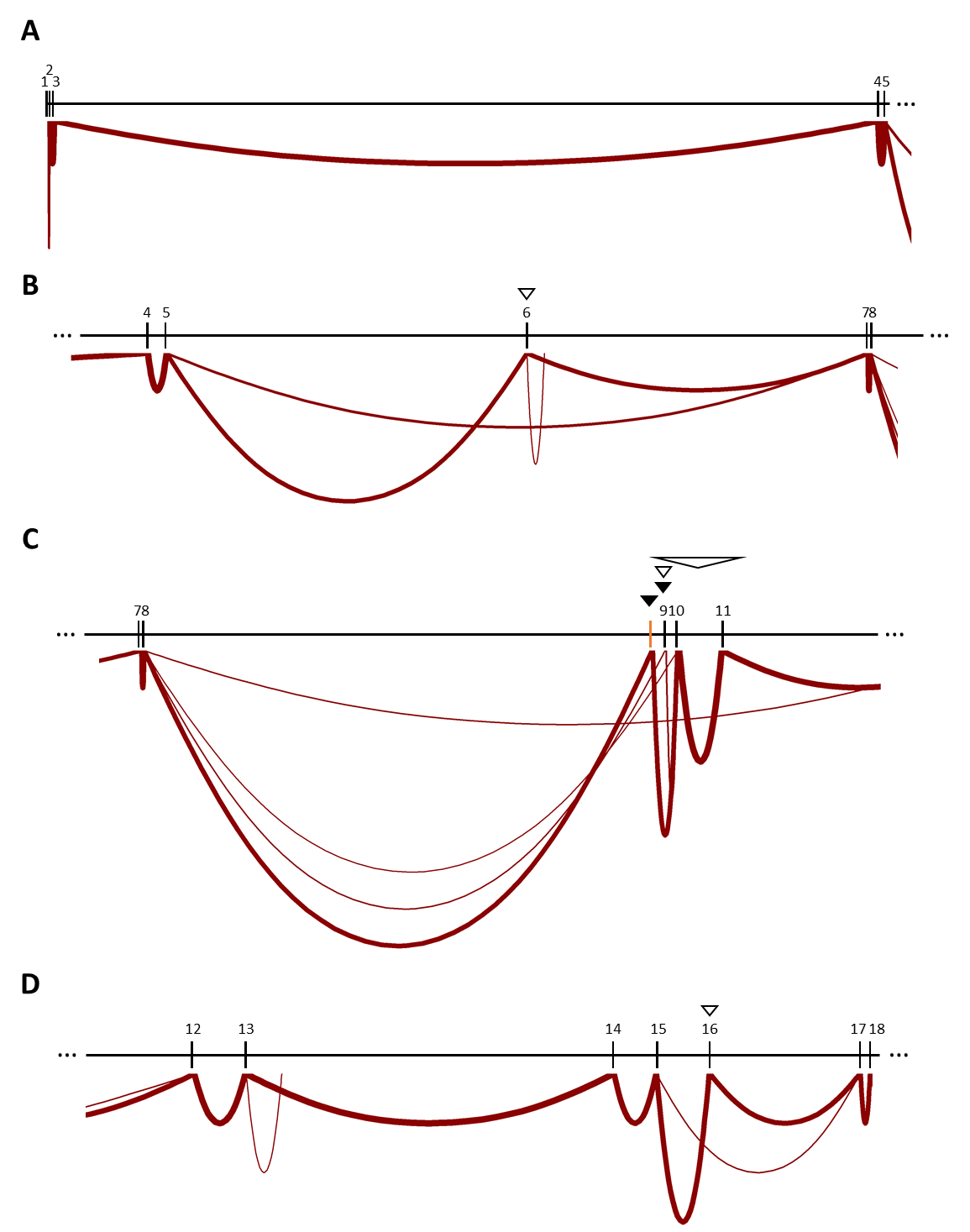
*

*Figure is continued on the next page.*

**
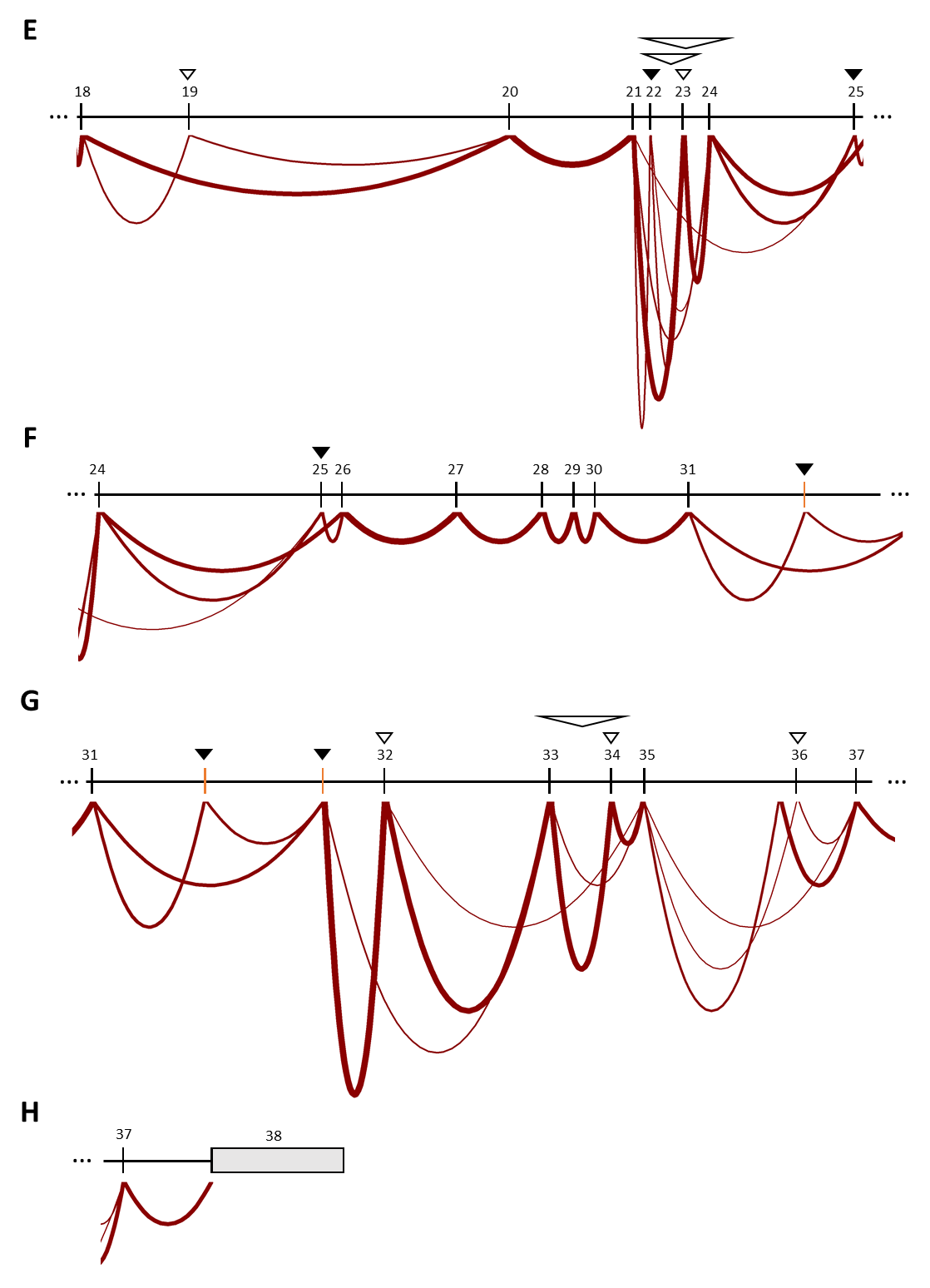
**

**Supplementary Fig. S3. Analysis of transcriptome sequencing data from 42 fibroblast-derived cDNA samples identified novel alternatively spliced *in-frame* exons of *COL25A1*. (A-H)** Sashimi plots show the number of sequence reads (indicated by thickness of lines) supporting detected *COL25A1* exon-exon junctions, based on merged data from 42 fibroblast cDNA samples. Only exon-exon junctions supported by at least 5 reads across the datasets are shown. Exons corresponding to *COL25A1* transcript variant NM_198721.4 (ENST00000399132.6) are represented by black boxes, while exons that are exclusively present in transcript variant NM_001256074.3 (ENST00000399127.5) are shown in orange. The predominant transcript variant expressed in fibroblasts is NM_001256074.3 (ENST00000399127.5). Introns are represented by horizontal black lines and the 3’ untranslated region by a grey box. Black arrowheads point to known and annotated alternatively spliced exons, and white arrowheads to novel (not yet annotated) alternatively spliced single or multiple exons. Alternative splicing of exon 9 was novel according to the transcript variant NM_198721.4 as identified by the exon 8-exon 10 junction **(C)**.

**SUPPLEMENTARY TABLES**

**Supplementary Table S1.** Sequence of oligonucleotides used in this work.

| ***COL25A1* primer sequences for variant validation and segregation analysis** | | | | |
| --- | --- | --- | --- | --- |
| **Template** | **Exon** | | **Direction** | **Sequence (5’ → 3’)** |
| DNA | 3 | | forward | GAC TCC ATC CCT CTA CAG TGC |
|  |  |  | reverse | TTA AAG CAA GTT GGG TCT GC |
|  | 4 | | forward | TGA TGG ACC TGA CTG AAA AG |
|  |  |  | reverse | ATT AGG AAG AAC AGA TGC CG |
|  | 23 | | forward | GCT ATT TTG GTG GTG GTT GC |
|  |  |  | reverse | GGA GGT AGG GGT GTT TCA GG |
|  | 33 | | forward | AAA GTG TGT GAA TGT AGG TGG G |
|  |  |  | reverse | GAA GGG TTA ACC GTG TCA GTG |
| ***COL25A1* primer sequences for transcript analysis (RT-PCR)** | | | | |
| **Template** | **Exon** | **Name** | **Direction** | **Sequence (5’ → 3’)** |
| cDNA | 2 | 2F | forward | CCC CTT CCA TTC ATC TGC T |
|  | 4 | 4R | reverse | TTA CCT CGT TTC CCT GGA G |
|  | 20 | 20F | forward | CAA GGA GAA CCA GGC TTA CC |
|  | 24 | 24R | reverse | TCT CAG TGG CTC CTT GAT CC |

Exon numbering is given according to *COL25A1* reference sequence NM_198721.4.

**Supplementary Table S2.** *In silico* pathogenicity and splice site predictions, minor allele frequency, and classification according to ACMG criteria of previously published and novel *COL25A1* variants.

| **Genomic position on chromosome 4 (hg38; NC_000004.12)** | **Nucleotide change**  **(NM_198721.4)** | **Exon** | **Amino acid alteration**  **(NP_942014.1)** | **gnomAD**  **(v4.1.0) MAF [%]** | **RGC Million Exome**  **MAF [%]** | **CADD**  **(>20)** | **REVEL**  **(>0.6)** | **Alpha-Missense**  **(>0.564)** | **SpliceAI**  **(≥0.2)** | **ACMG classification** | **Reference** |
| --- | --- | --- | --- | --- | --- | --- | --- | --- | --- | --- | --- |
| g.109,262,132_  109,332,470del | ─ | 1-3 | p.0? | absent | n. a. | n. a. | n. a. | n. a. | n. a. | likely pathogenic  (2C) | this study (subject 4) |
| g.109,300,583C>G | c.367G>C | 3 | r.300_367del;  p.(Ser101Profs*7) | absent | absent | 37 | 0.868 | 0.940 | DL: 0.97 | likely pathogenic  (PVS1 [RNA], PM2) | this study (subject 3) |
| g.108,931,745_  109,055,301del | ─ | 4-11 | p.? | absent | n. a. | n. a. | n. a. | n. a. | n. a. | likely pathogenic  (2E) | [4] |
| g.109,050,165G>A | c.382C>T | 4 | p.(Arg128*) | 0.000684 | 0.0006691 | 39 | n. a. | n. a. | no impact | pathogenic  (PVS1, PM2_sup, PM3) | this study (subject 7) |
| g.108,940,538del | c.672+1del | In10 | r.?; p.? | absent | absent | 26.1 | n. a. | n. a. | DL: 0.99 | likely pathogenic  (PVS1, PM2, PM3_sup) | [5] |
| g.108,940,538C>T | c.672+1G>A | In10 | r.?; p.? | 0.0004341 | 0.0006692 | 32 | n. a. | n. a. | DL: 0.99 | likely pathogenic  (PVS1, PM2_sup, PM3_sup) | [5] |
| g.108,863,327C>T | c.1144G>A | 21 | p.(Gly382Arg) | 0.0003719 | 0.0005475 | 26.5 | 0.997 | 0.962 | no impact | likely pathogenic  (PM2_sup, PM3, PP3_strong) | [4] |
| g.108,860,971C>T | c.1198G>A | 23 | p.(Gly400Arg) | absent | absent | 31 | 0.974 | 0.976 | AG: 0.28 | likely pathogenic  (PM2, PM3_sup, PP3_strong) | [5] |
| g.108,860,971C>A | c.1198G>T | 23 | p.(Gly400Trp) | absent | absent | 32 | 0.957 | 0.964 | no impact | likely pathogenic  (PM2, PM3, PP3_strong) | this study (subject 3) |
| g.108,846,204T>C | c.1450A>G | 28 | p.(Lys484Glu) | absent | 0.0000608 | 26 | 0.773 | 0.218 | no impact | VUS  (PM2_sup, PM3, PP3_mod) | [5] |
| g.108,846,165C>A | c.1489G>T | 28 | p.(Gly497*) | absent | absent | 42 | n. a. | n. a. | no impact | pathogenic  (PVS1, PM2, PM3) | [4] |
| g.108,844,552del | c.1598del | 30 | p.(Pro533Hisfs*77) | absent | absent | 34 | n. a. | n. a. | no impact | pathogenic  (PVS1, PM2, PM3) | this study (subject 5) |
| g.108,827,169C>T | c.1730G>A | 33 | p.(Gly577Glu) | absent | absent | 32 | 0.984 | 0.969 | no impact | likely pathogenic  (PM2, PM3, PP3_strong) | this study  (subjects 1 and 2) |
| g.108,697,001_  108,827,000del | ─ | 33-38 | p.? | absent | n. a. | n. a. | n. a. | n. a. | n. a. | likely pathogenic  (2D) | this study (subject 6) |

Worldwide allele frequency of *COL25A1* variants in the gnomAD database v4.1.0 [6] and the Regeneron Genetics Center (RGC) Million Exome data [7] is given. The functional impact of biallelic *COL25A1* variants was predicted by the Combined Annotation Dependent Depletion (CADD) tool, the Rare Exome Variant Ensemble Learner (REVEL) scoring system, and the deep learning model AlphaMissense. CADD is a framework that integrates multiple annotations in one metric by contrasting variants that survived natural selection with simulated mutations. Reported CADD scores are phred-like rank scores based on the rank of that variant’s score among all possible single nucleotide variants of hg19, with 10 corresponding to the top 10%, 20 at the top 1%, and 30 at the top 0.1%. The larger the score the more likely the variant has deleterious effects; the score range observed here is strongly supportive of pathogenicity, with all observed variants ranking above ~99% of all variants in a typical genome and scoring similarly to variants reported in ClinVar as pathogenic (~85% of which scores >15) [8]. REVEL is an ensemble method predicting the pathogenicity of missense variants with a strength for distinguishing pathogenic from rare neutral variants with a score ranging from 0-1, with a pathogenicity threshold of ≥0.6 [9]. AlphaMissense is a computational tool predicting missense variant pathogenicity by combining AlphaFold-based structural information and evolutionary conservation. The score ranges from 0-1, with a pathogenicity threshold of ≥0.564 [10]. A possible effect of variants on splicing was analyzed using the open-source deep learning splicing prediction algorithm SpliceAI. The score ranges from 0-1, with a cutoff of ≥0.2 [11]. *COL25A1* variants were classified according to the standardized guidelines of the *American College of Medical Genetics and Genomics* (ACMG) [12]. AG, splice site acceptor gain; DL, splice site donor loss; In, intron; MAF, minor allele frequency; mod, moderate; n. a., not applicable; sup, supporting; VUS, variant of unknown significance.

**Supplementary Table S3.** *In silico* splice site predictions for the *COL25A1* variants c.367G>C and c.1198G>T.

|  | **splice donor site in intron 3** | | **splice acceptor site in intron 22** | |
| --- | --- | --- | --- | --- |
|  | **reference sequence** | **sequence with c.367G>C variant** | **reference sequence** | **sequence with c.1198G>T variant** |
| **SpliceSiteFinder-like (range 0-100)** | 82.5 | ─ | ─ | ─ |
| **NNSPLICE (range 0-1)** | 0.9 | 0.6 | ─ | ─ |
| **MaxEntScan (range 0-16)** | 8.6 | ─ | ─ | ─ |
| **GeneSplicer (range 0-21)** | 3.3 | ─ | ─ | ─ |

Splice donor and acceptor splice site prediction scores were calculated for the *COL25A1* reference sequence (NM_198721.4) and the sequence with the c.367G>C or the c.1198G>T variant using Alamut Visual v2.15 (SOPHiA GENETICS, Lausanne, Switzerland) which includes the following splice site prediction algorithms: SpliceSiteFinder [13, 14], NNSPLICE 0.9 version [15], MaxEntScan [16], and GeneSplicer [17]. High and low scores indicate strong and weak splice sites, respectively. ̶ : splice site not recognized.

**SUPPLEMENTARY REFERENCES**

1. Li H, Durbin R. Fast and accurate long-read alignment with Burrows-Wheeler transform. Bioinformatics. 2010;26(5):589-95.

2. McKenna A, Hanna M, Banks E, Sivachenko A, Cibulskis K, Kernytsky A, et al. The Genome Analysis Toolkit: a MapReduce framework for analyzing next-generation DNA sequencing data. Genome Res. 2010;20(9):1297-303.

3. Wang K, Li M, Hakonarson H. ANNOVAR: functional annotation of genetic variants from high-throughput sequencing data. Nucleic Acids Res. 2010;38(16):e164.

4. Shinwari JM, Khan A, Awad S, Shinwari Z, Alaiya A, Alanazi M, et al. Recessive mutations in COL25A1 are a cause of congenital cranial dysinnervation disorder. Am J Hum Genet. 2015;96(1):147-52.

5. Natera-de Benito D, Jurgens JA, Yeung A, Zaharieva IT, Manzur A, DiTroia SP, et al. Recessive variants in COL25A1 gene as novel cause of arthrogryposis multiplex congenita with ocular congenital cranial dysinnervation disorder. Hum Mutat. 2022;43(4):487-98.

6. Chen S, Francioli LC, Goodrich JK, Collins RL, Kanai M, Wang Q, et al. A genomic mutational constraint map using variation in 76,156 human genomes. Nature. 2024;625(7993):92-100.

7. Sun KY, Bai X, Chen S, Bao S, Zhang C, Kapoor M, et al. A deep catalogue of protein-coding variation in 983,578 individuals. Nature. 2024;631(8021):583-92.

8. Kircher M, Witten DM, Jain P, O'Roak BJ, Cooper GM, Shendure J. A general framework for estimating the relative pathogenicity of human genetic variants. Nat Genet. 2014;46(3):310-5.

9. Ioannidis NM, Rothstein JH, Pejaver V, Middha S, McDonnell SK, Baheti S, et al. REVEL: An Ensemble Method for Predicting the Pathogenicity of Rare Missense Variants. American Journal of Human Genetics. 2016;99(4):877-85.

10. Cheng J, Novati G, Pan J, Bycroft C, Zemgulyte A, Applebaum T, et al. Accurate proteome-wide missense variant effect prediction with AlphaMissense. Science. 2023;381(6664):eadg7492.

11. Jaganathan K, Kyriazopoulou Panagiotopoulou S, McRae JF, Darbandi SF, Knowles D, Li YI, et al. Predicting Splicing from Primary Sequence with Deep Learning. Cell. 2019;176(3):535-48 e24.

12. Richards S, Aziz N, Bale S, Bick D, Das S, Gastier-Foster J, et al. Standards and guidelines for the interpretation of sequence variants: a joint consensus recommendation of the American College of Medical Genetics and Genomics and the Association for Molecular Pathology. Genet Med. 2015;17(5):405-23.

13. Zhang MQ. Statistical features of human exons and their flanking regions. Hum Mol Genet. 1998;7(5):919-32.

14. Shapiro MB, Senapathy P. RNA splice junctions of different classes of eukaryotes: sequence statistics and functional implications in gene expression. Nucleic Acids Res. 1987;15(17):7155-74.

15. Reese MG, Eeckman FH, Kulp D, Haussler D. Improved splice site detection in Genie. J Comput Biol. 1997;4(3):311-23.

16. Yeo G, Burge CB. Maximum entropy modeling of short sequence motifs with applications to RNA splicing signals. J Comput Biol. 2004;11(2-3):377-94.

17. Pertea M, Lin X, Salzberg SL. GeneSplicer: a new computational method for splice site prediction. Nucleic Acids Res. 2001;29(5):1185-90.
